# Effect of maternal *Helicobacter Pylori* infection on birth weight in an urban community in Uganda

**DOI:** 10.1186/s12884-016-0950-8

**Published:** 2016-07-13

**Authors:** Ronald Wanyama, Mike N. Kagawa, Kenneth C. Opio, Rhona K. Baingana

**Affiliations:** Department of Biochemistry, Faculty of Medicine, Gulu University, P.O. Box 166, Gulu, Uganda; Department of Obstetrics & Gynecology, College of Health Sciences, Makerere University, P.O. Box 7062, Kampala, Uganda; Department of Internal Medicine, College of Health Sciences, Makerere University, P.O. Box 7062, Kampala, Uganda; Department of Biochemistry and Sports Science, School of Biological Sciences, Makerere University, P.O. Box 7062, Kampala, Uganda

**Keywords:** *H. pylori* infection, Pregnancy, Birth weight, Gestation weight gain, Kampala Uganda

## Abstract

**Background:**

*Helicobacter pylori,* a widespread infection particularly in developing countries has been associated with many adverse effects during pregnancy including hyperemesis gravidarum, neural tube defects in newborns, intrauterine fetal growth restriction and miscarriage. We sought to document the effects of *H. pylori* infection on birth weight in a low-income setting in Kampala, Uganda.

**Methods:**

This was a prospective cohort study conducted in Kampala between May 2012 and May 2013. The participants were *H. pylori* positive and *H. pylori* negative HIV negative primigravidae and secundigravidae. Recruitment was at ≤18 gestation weeks and follow up assessments were carried out at 26 and 36 gestation weeks and soon after delivery. *H. pylori* infection was determined using *H. pylori* stool antigen test. Maternal weight and height were measured, and body mass index (BMI) and gestational weight gain were calculated. Only term and live babies were considered. Low birth weight (LBW) was defined as a birth weight of <2500 gram.

**Results:**

A total of 221 participants were enrolled with mean ± standard deviation (SD) age of 20.9 ± 2.7 years. The mean ± SD gestation age at delivery was 39.4 ± 1.0 weeks. Primigravidae were 61.5 % (*n* = 188) and 52.9 % (*n* = 117) of the participants were positive for *H. pylori* infection. Low pre-pregnancy BMI (<18.5 kg/m^2^) was recorded in 14.6 % (*n* = 28) while 38 % (*n* = 73) had a height <156 cm at recruitment. Of the infants born to the participants, 13.6 % (*n* = 26) had low birth weight (<2500 gram).

Independent predictors for LBW were the mother being positive for *H. pylori* infection (odds ratio, OR, 3.6, 95 % CI 1.1 – 11.5; *P* = 0.031) maternal height at recruitment <156 cm (OR 3.4, 95 % CI 1.4–8.2; *P* = 0.008) and maternal weight gain rates <0.3 kg/week during the 2^nd^ and 3^rd^ trimesters (OR 3.8, 95 % CI 1.0–14.1; *P* = 0.044).

**Conclusion:**

*H. pylori* infection is associated with LBW among primigravidae and secundigravidae in Kampala, Uganda.

## Background

*Helicobacter pylori* (*H. pylori*) infection is the most common bacterial infection worldwide. Almost half of people in developed countries and three-quarters of people in developing countries are infected with *H. pylori* [1, 2]. Although many infected individuals are asymptomatic, *H. pylori* is an important health problem. *H. pylori* infection has been recognized as a major cause of various gastroduodenal diseases, such as chronic gastritis, peptic ulcer disease, and gastric cancer [3]. In Uganda the prevalence was 74 % in patients with dyspepsia referred for endoscopy and 86 % in patients with cancer and benign tumors [4, 5].

A high prevalence of *H. pylori* has been observed among pregnant women. In Uganda, Baingana et al. recently found 60 % prevalence of *H. pylori* infection among pregnant women [6]. Studies in Sudan, Mexico and Chile found prevalence rate of 69.8 %, 52.2 % and 68.6 % in respectively [7–9]. *H. pylori* infection in pregnancy is associated with many adverse effects, such as extreme, persistent nausea and vomiting (hyperemesis gravidarum) [8, 10], neural tube defects in newborns, pre-eclampsia with intrauterine fetal growth restriction and miscarriage, and thrombocytopenia [11–14]. Furthermore, pregnant women infected with *H. pylori* infection are at increased risk of anemia [15, 16].

Pregnancy is a physiological condition in which a marked increase in body weight occurs over a short period. An optimum weight gain over the course of pregnancy, as recommended by the Institute of Medicine (IOM, 2009), is one that produces a healthy newborn. Optimum weight gain also provides sufficient postpartum maternal fat stores to support lactation without increasing obesity risk [17]. Gestational weight gains below the IOM recommendation are common in developing countries [18]. There is evidence to show that maternal pre-pregnancy weight and the weight gained during pregnancy influence birth weight [19]. Inadequate gestational weight gain increases the risk of preterm delivery and low birth weight (LBW) infants [20, 21].

Birth weight is an important determinant of an infant’s well-being. Infants born with LBW are at increased risk of morbidity and mortality from infectious disease, and suffer underweight, stunting or wasting beginning in the neonatal period through childhood [22]. There is evidence that adults born with LBW face an increased risk of chronic diseases including high blood pressure, type II diabetes mellitus, coronary heart disease and stroke in adulthood [23]. In Uganda, the prevalence of LBW, at 10.5 %, is high [24].

An association between *H. pylori* and LBW has been suggested [13, 14]. However, there is limited data on association between *H. pylori* and birth weight especially in developing countries. The objective of this study was to establish the association between *H. pylori* infection and birth weight.

## Methods

### Study design and site

This was a prospective cohort study in which pregnant women were followed from the time of recruitment up to delivery between May 2012 and May 2013. The study was conducted at the antenatal clinic of Kawempe Health Centre IV. The Health Centre is supported by the Ministry of Health, Uganda and the services in the antenatal clinic are free to the public. This clinic serves a densely-populated, low-income area in Kawempe, one of the five Divisions forming Kampala District in Uganda. The Division is located in the Northern part of Kampala District.

### Study population

Pregnant women attending antenatal at Kawempe Health Centre constituted the study population. The target population was HIV negative primigravidae and secundigravidae.

### Sample size

We used online openEpi software, http://www.openepi.com, based on Kelsey Lesley (1996) to calculate sample size. In the formula we used a confidence level of 95 %, power of 80 %, ratio of *H. pylori* positive to *H. pylori* negative of one. Furthermore, in the formula we used 18 and 35 as the percentages of unexposed and exposed participants with outcome of interest according to Eslick [14]. The sample was divided two groups (exposed and unexposed). The exposed group (141) comprised of those who tested positive for *H. pylori* infection while the unexposed group (90) comprised who tested negative for *H. pylori* infection. We anticipated 5 % lost to follow up.

### Recruitment and follow up

A consecutive sampling procedure was used to select participants who met the selection criteria until the sample size was achieved. The participants were chosen as they got registered at the antenatal clinic. Written consent for each eligible participant was sought after clear information being given about the study objectives, procedures and benefits. In Uganda HIV testing for pregnant women is mandatory and is always done on the day of the first visit antenatal clinic. The study participants were recruited as informed volunteers at 12–18 weeks of gestation based on the reported last menstrual period and the experienced midwife’s examination. Follow up assessments were carried at 26 and 36 weeks of gestation. This study only considered term neonates, that is, babies who were delivered after 37 weeks of gestation.

The study participants were included in our cohort based on the following criteria; between 18–35 years of age, pregnant for the first or second time, HIV negative, carrying singleton pregnancy, free of any systemic illness such as hypertension, active peptic ulcers, diabetes mellitus or genetic abnormality, for example, sickle cell disease, between gestation weeks 12–18 at the time of recruitment. However, some of the pregnant women were excluded from this study based on the following criteria; not able to recall their pre-pregnancy weight, not able to schedule their return visits, not able to speak and/or hear, mentally ill, history of drug or alcohol abuse Based on the set exclusion criteria, a total of 56 women excluded from this study. Fourteen of them could not adhere to the scheduled return visits, 2 were sickle cell patients, 4 were alcohol abusers, 28 could not recall their pre-pregnancy weight, 6 had active peptic ulcers and 2 were carrying twins.

### Data collection and determination of nutritional status

During participant’s interview, demographic data including social, behavioral and medical history were collected in structured questionnaires. Nutritional status of each participant was assessed using anthropometric parameters. Anthropometric measurements were carried out in a closed room when the participant was barefoot and wearing light clothing. Body weight was measured using an adult a portable beam scale with 150 kg capacity divided into 0.5 kg increments (Gmbh & co.kg, Germany model 7621019009). Height was determined with the individual barefoot and in an orthostatic position with the aid of a portable stadiometer consisting of a non-extendable 2 meter measuring tape divided into 0.1 cm increments. Body weight and height were measured twice for every participant and the average of the readings was considered as the participant’s weight and height respectively. Each participant’s BMI was calculated using the following formula: BMI = body weight (kg) divided by [height (m)]^2^. The BMI was categorized as follows; underweight (<18.5 kg/m^2^), normal weight (18.5–24.9 kg/m^2^), overweight (25.0–29.9 kg/m^2^), obese (≥30.0 kg/m^2^) [25]. The nutritional status of infants was assessed from the birth weight measurements. Birth weight was measured by baby weight scale (Fazzini) within 24 h after birth. A neonate was considered LBW if it weighed less than 2500 grams.

### Stool collection and testing for *H. Pylori* infection

After clear instructions on how to collect stool, each participant was given clean tissue paper on which to deposit the stool. After, she had to immediately transfer a stool sample into stool collection bottle using the scooper which was part of the bottle top. This was done in the antenatal clinic toilet. Stool samples were immediately placed in a cool box with ice packs. The samples were transported everyday from Kawempe Health Centre to the laboratory (~3 km) and stored in a −20 ° C freezer until analysis was carried out. *H. pylori* stool antigen test, i-Chek cassettes (Chem-Labs Limited, Nairobi, Kenya) were used to analyze the stool samples. It is a rapid one-step chromatographic immunoassay that utilizes a combination of anti-*H. pylori* antibodies and anti-mouse IgG. Instructions given by the manufacturer were followed. Approximately 100 μl of stool of completely thawed stool was brought into the sample diluent tube and vortexed for fifteen seconds. Three drops of the diluted sample were applied to the test and the result was read after fifteen minutes. The results were reported as positive or negative on the basis of the manufacturer’s guidelines. A procedural control was included with each test.

### Data analysis

Data were analyzed using Statistical Package for Social Sciences (SPSS) V.16.0 (SPSS Inc., Chicago, IL, USA). Social, demographic and measurement parameters were summarized into frequencies and mean ± standard deviation (SD). The outcome variable was birth weight while the predictor variables were *H. pylori* infection, rates of gestation weight gains pre-pregnancy weight, pre-pregnancy BMI, parity and maternal height. Continuous data were checked for normality. Tests for the significance of association were made using the Pearson chi-square (*χ*^*2*^*)* test for categorical variables and independent sample *t* test for continuous variables. Factors associated with birth weight were determined with logistic regression. Factors associated with birth weight with *P* values <0.05 during univariate analysis were considered for multivariate analysis using logistic regression to determine factors independently associated with birth weight. Odds ratio (OR) and 95 % confidence interval (CI) were reported. At multivariate analysis, statistical significance was determined if *p* < 0.05.

## Results

Table 1 summarizes the overall socio-demographic characteristics of the study participants. Two hundred 221 HIV-negative pregnant women were enrolled into the study. 26 of the 221 were lost to follow up and of 26, 20 were negative for *H. pylori* infection. All the 221 enrolled were used to calculate the prevalence of *H. pylori* infection among the participants. The prevalence *H. pylori* infection was 52.9 % (117/221). However, data from only 192 participants was used to perform all the other analyses because two of the participants lost their pregnancies at 22 and 25 weeks of gestation, one delivered before 37 weeks of gestation. Birth weight of one baby born to a participant who completed the study was not captured as it was still birth. 61.5 % (188/192) of the participants were pregnant for the first time. 87.5 % (168/192) of the participants were married, 9.9 % (19/192) were single and the rest were either divorced/separated or widowed. Only 1.0 % (2/192) and 5.2 % (10/192) were smokers and were taking alcohol respectively. The majority, 64.6 % (124/192), of the participants had acquired secondary education. The majority of the participants 78.1 % (144/192) were housewives and only 19.8 % (38/192) were employed. Underweight (BMI <18.5 kg/m^2^) was recorded in 14.6 % (28/192) of the participants. 38.0 % (73/192) of the participants had height <156 cm and 65.6 % (126/192) were less than 21 years of age. Of the infants born to participants, 52.6 % (101/192) were females and 13.6 % (26/191) had low birth weight (<2500 g). From Tables 1 and 2, we observe that there were no differences in the socio-demographic variables between the participants with *H. Pylori* infection and those without *H. Pylori* infection except for LBW (*P* = 0.002).

**Table 1:** Comparison of categorical socio-demographic characteristics by *H. pylori* status

Variable (*n* = 192)	*H. pylori* positive	*H. pylori* negative	*P* value
Parity			
Primigravidae	70	48	
Secundigravidae	40	34	0.473^*^
Marital status			
Married	94	74	
Single	12	7	
Widowed/Divorced	4	1	0.445^*^
Maternal pre-pregnancy BMI (kg/m^2^)			
Underweight (<18.5)	15	13	
Normal weight (18.5–24.9)	80	63	
Overweight (25.0–29.9)	15	6	0.372^*^
Sex of the baby			
Female	56	44	
Male	54	38	0.706
Birth weight (grams)^a^			
<2500	22	4	
≥2500	87	78	0.002^*^
Maternal pre-pregnancy height (cm)			
<156	42	31	
≥156	68	51	0.958^*^
Maternal age (years)			
<21	70	56	
≥21	40	26	0.502^*^
Occupation	79	65	
House wife			
Peasant	1	9	
Employee	23	15	
Student	7	2	0.561^*^
Smoking			
Yes	0	2	
No	110	80	0.100^*^
Building type			
Permanent	108	82	
Temporary	2	0	0.518^*^
Alcohol			
Yes	5	5	
No	105	77	0.632^*^
Water source			
Tap	87	64	
Borehole	6	5	
Protected well	17	11	
Unprotected well	0	1	
Tank (harvested rain water)	0	1	0.490^*^
Education level			
Low (No education to primary 7)	22	20	
Medium (Secondary level)	72	52	
High (tertiary education)	16	10	0.114^*^
Household monthly income ($)			
Low income (<100)	56	38	
Medium income (101–250)	46	41	
High income (>250)	8	3	0.374^*^

^*^ *P* valve for the Pearson chi-square for the difference between *H. pylori* positive and *H. pylori* negative; *n* is number, ^a^one still birth was not weighed

**Table 2:** Comparison of continuous socio-demographic characteristics by *H. pylori* status

Variable (*n* = 192)	*H. pylori* positive	*H. pylori* negative	*P* value
Maternal age (years)	21.1	20.8	0.481^**^
Mean household size	3.0	2.8	0.462^**^
Mean maternal recruitment BMI (kg/m^2^)	21.6	21.2	0.352^**^
Mean maternal height (cm)	157.5	157.2	0.727^**^

^**^ *P* valve for the independent sample *t* test and *n* is number

Table 3 shows the means ± standard deviations (SD) and ranges for the selected variables and the difference between *H. pylori* positive and *H. pylori* negative participants. The participants had a mean ± SD (kg/week) rate of weight gain of 0.30 ± 0.11 with a range of 0.08–0.75 and there was a difference in the rates of weight gain between *H. pylori* positive and *H. pylori* negative participants (*P* < 0.001). The mean ± SD gestation age at delivery was 39.4 ± 1.0 with a range of 37 – 42 weeks. There was no difference in mean gestation age at delivery between *H. pylori* positive and *H. pylori* negative participants (*P* = 0.494). The mean ± SD birth weight was 2922 ± 476 g with a range of 1700–4400 g and the mean birth weight of babies born to *H. pylori* positive mothers was significantly different from that of babies born to *H. pylori* negative participants (*P* < 0.001). The mean ± SD (range) pre-pregnancy weight, pre-pregnancy BMI and maternal height were 53.1 ± 7.7 kg (37–76 kg), 21.5 ± 2.7 kg/m^2^ (15.0–29.4 kg/m^2^) and 157.4 ± 5.8 cm (142.0–173.1 cm) respectively. The mean pre-pregnancy weight, pre-pregnancy BMI and maternal height of *H. pylori* positive participants were not significantly different from those of *H. pylori* negative participants.

**Table 3:** Mean ± SD values selected variables in relation to *H. pylori* infection status

Variable	Mean ± SD by *H. Pylori* Status		Range	*P* valve
	*H. pylori* -ve (*n* = 82)	*H. pylori* + ve (*n* = 110)		
Rate of weight gain (kg/week)	0.33 ± 0.11	0.26 ± 0.10	0.08–0.75	<0.001
Gestational age (weeks)	39.4 ± 1.1	39.3 ± 1.0	37.0–42.0	0.494
Birth weight (g)	3245 ± 407	2681 ± 370	1700–4400	<0.001
Pre-pregnancy BMI (kg/m^2^)	21.2 ± 2.5	21.6 ± 2.9	15.0–29.4	0.35
Pre-pregnancy weight (kg)	52.6 ± 7.0	53.5 ± 8.1	37–76	0.392
Maternal height (cm)	157.3 ± 5.9	157.5 ± 5.9	142.0–173.1	0.727

### Factors associated with low birth weight

For logistic regression, low birth weight (BW <2500 grams) was used as the outcome variable. The covariates were *H. pylori* status, maternal gestation rate of weight gain, maternal height, and parity (Table 4). However, the independent predictors for LBW were the mother being positive for *H. pylori* infection (odds ratio, OR, 3.6, 95 % CI 1.1–11.5; *P* = 0.031), maternal height at recruitment <156 cm (OR 3.4, 95 % CI 1.4–8.2; *P* = 0.008) and maternal gestation weight gain rates <0.3 kg/week during the 2^nd^ and 3^rd^ trimesters (OR 3.8, 95 % CI 1.0–14.1; *P* = 0.044) (Table 5).

**Table 4:** Factor associated with low birth weight at univariate analysis

Variable	Odds ratio for LBW	*P* valve (95 % CI)
Maternal rate of weight gain <0.3 kg/week	5.1	0.011 (1.5, 17.5)
Maternal height <156 cm	3.0	0.012 (1.3, 6.8)
Primigravidae	1.4	0.048 (1.1, 3.4)
*H. pylori* infection	4.9	0.005 (1.6, 14.8)

**Table 5:** Factor independently associated with low birth weight

Variable	Odds ratio for LBW	*P* valve (95 % CI)
*H. pylori* infection	3.6	0.031 (1.1, 11.5)
Maternal rate of weight gain <0.3 kg/week	3.8	0.044 (1.0, 14.1)
Maternal height <156 cm	3.4	0.008 (1.4, 8.2)
Primigravidae	1.08	0.895 (0.34, 3.4)

Variables entered on step 1: *H. pylori* infection, maternal rate of weight gain, maternal height, and parity

## Discussion

The prevalence of *H. pylori* infection on the basis of presence of *H. pylori* stool antigens in this population of primigravidae and secundigravidae was 52.9 %. This is within the range expected considering our country’s socioeconomic level and prevalence of the infection among pregnant women attending antenatal care clinic in the same health centre in Kampala [2, 6]. The high rate of *H. pylori* infection in this study mirrors the observation of 69.8 %, 68.5 % and 52.2 % rate of *H. pylori* infection among pregnant women in neighboring Sudan, Chile and rural Mexico respectively [7–9]. Higher prevalence, 74 % and 88 %, of *H. pylori* infection was reported among pregnant women at the US–Mexico border and Egypt respectively [26, 27]. Our study comprised on primigravidae and secundigravidae with the a mean ± SD age of 20.9 ± 2.7 years and there is evidence that prevalence of *H. pylori* infection increase with increasing age and number of deliveries [9].

Infants born from *H. pylori* positive women had a significantly lower mean ± SD birth weight (2681 ± 370 g) than those born from *H. pylori* negative women (3245 ± 407 g) *P* < 0.001. This perfectly agrees with the findings of the study done in Turkey by Mulayim et al. [16]. Similarly infants born to primigravidae had a significantly lower mean ± SD birth weight (2791 ± 422 g) than those born to secundigravidae (3210 ± 465 g) *P* < 0.001. This finding is in agreement with the findings of Chiba et al. [28]. However, there were no differences in the LBW prevalence between infants born to primigravidae and those born to secundigravidae (*P* = 0.70). This finding is in contrast with the findings of Chiba and his colleagues who found out that the LBW rate was significantly higher in primigravidae than in multigravidae females. This difference could be because our study population was homogenous in most of the socio-demographic and nutritional characteristics to detect significant association.

The prevalence of LBW in this study population was 13.6 % and this was slightly higher than the National LBW prevalence (10.5 %) but mirrors the 14 % which has been reported in parts of central Uganda where this study was conducted [24]. We believe LBW in this urban population was higher because of the nature of the population we studied. In the present study, only those women pregnant for the 1^st^ or 2^nd^ time with mean ± SD age was 20.9 ± 2.7 years were included while women of urban and rural communities irrespective of gravidae and age were studied in the National survey [24]. A good number of our participants (38.5 %) were pregnant for the 2^nd^ time with mean ± SD age of 21.7 ± 3.2 years. There is a body of evidence which show that first born and second born babies born to adolescents have higher chances of being born LBW [29]. There is also current evidence that age <20 years increases the risk of giving birth to LBW baby [30].

In this study we found *H. pylori* infection to be an independent predictor of low birth weight (OR = 3.6, 95 % CI: 1.1 – 11.5; *P* = 0.031). Our finding is in agreement with the finding of Eslick et al. and Mulayim et al. [14, 16]. Eslick et al. (2002) observed for the first time association between *H. pylori* infection and low birth weight, in particular they showed that intrauterine growth restriction was more common in *H. pylori* positive women (13.5 %) than in negative mothers (6.0 %) (OR = 2.41; 95 % CI: 1.14–5.08; *P* = 0.018). Mulayim et al. observed that pregnant women with *H. pylori* infection delivered neonates with a significantly lower birth-weight compared to mothers without the infection. Furthermore, in animal models, it has been reported that *H. pylori* infected mice showed a decrease in implantation rates, and their offspring were of low birth weight [31]. However, in another experimental mice model study these results were not confirmed [32]. The mechanism(s) by which *H. pylori* infection causes fetal growth retardation is/are not very clear but several have been suggested. *H. pylori* has been linked with an increase in symptoms including dyspepsia, nausea or vomiting [8], because of underlying undiagnosed peptic ulcer disease, which in turn may affect maternal gastric absorption and therefore impair fetal growth. In humans, *H. pylori* infection has also been associated with anemia [33] and maternal anemia associated to *H. pylori* infection may lead to fetal growth restriction. In the study by Jasem et al., [33], fetal growth retardation could have been due to maternal anemia since all anemic pregnant women were in the *H. pylori* positive group. Fetal growth retardation may be due to either maternal or feto-placental causes. Feto-placental causes include infections and other placental pathologies [34]. It has been demonstrated that anti-CagA antibodies cross-react in vitro with placental tissue reducing its invasiveness ability [35] and the consequent abnormal placentation could lead to fetal growth restriction [34]. However, Cardaropoli et al. found a strong association between *H. pylori* infection and fetal growth retardation in preeclamptic pregnancies, while there was no association between *H. pylori* and idiopathic fetal growth retardation [12].

The pattern of maternal weight gain during pregnancy is an important determinant of fetal growth [17]. This study found out that primigravidae had a higher mean ± SD rate of weight gain (0.33 ± 0.11 kg/week) than secundigravidae (0.27 ± 0.10 kg/week) *P* < 0.001. This finding is in agreement with recent findings of Lumbanrajaa et al.*,* [36]. This present study also found rates of gestation weight gain rates <0.30 kg/week during the 2^nd^ and 3^rd^ trimesters to be an independent predictor of low birth weight (OR = 3.8, 95 % CI 1.0–14.1; *P* = 0.044). This finding of ours agrees with several other studies which have reported that birth weight has positive correlations with maternal gestation weight [37–39].

There was no difference in mean ± SD maternal height between *H. pylori* positive (157.54 ± 5.92 cm) and *H. pylori* negative (157.25 ± 5.92 cm) (*P* = 0.73). However, maternal height less than 156 cm was another important parameter which independently increased the risk for LBW (OR = 3.4, 95 % CI 1.4–8.2; *P* = 0.008). Our cut off point of 156 cm agrees well with other investigators in Sudan, Brazil and Bangladesh who also found that maternal height below 156 cm increases the risk for LBW and child mortality [30, 40, 41]. Elshibly et al. [41] found maternal height <156 cm to increase LBW (AUC 0.591, 95 % CI 0.560–0.622; *P =* 0.003). This confirms the value of maternal height as a predictor of childhood morbidity and mortality.

The strength of our study lies in the fact it was a prospective cohort and we were able to control some of the known risk factors for LBW such as preterm delivery, chronic and genetic diseases. We also included a homogenous population and we are able to attribute the birth weights to *H. pylori* infection. We also included only children delivered after 37 weeks of gestation so premature babies were not part of our cohort. However, this current study had some limitations. We did not collect data of all the risk factors for LBW, for example, anemia, level of physical activity during pregnancy, number of antenatal visits, previous poor pregnancy outcome for secundigravidae and complications which occurred during pregnancy and labor neither did we consider other infections, such as malaria and helmith, which are endemic in the area and were previously reported as a cause of low LBW [42, 43].

## Conclusion

In conclusion, the most important finding of this study was that *H. pylori* infected pregnant women showed a significantly a higher risk of giving birth to LBW babies. In addition, primigravidae and secundigravidae with less a height less than 156 cm had a higher risk of giving birth to LBW babies. Irrespective of Maternal *H. pylori* infection status, maternal height and gestation rates of weight gain remained the most important factors for LBW.

### Recommendations

We recommend *H. pylori* infection screening for women of child bearing age. Those found positive for *H. pylori* infection should be treated before they get pregnant since the available drug regimes for treatment of *H. pylori* are not safe in pregnancy. Pregnant women should also be routinely counseled and monitored for gestation weight gain to ensure adequate weight which results into a normal birth weight baby.

## Abbreviations

BMI, body mass index; *H. pylori, Helicobacter pylori*; HIV, Human immunodeficiency virus; IOM, Institute of Medicine; LBW, low birth weight; OR, odds ratio; SD, standard deviation; SPSS, statistical package for social Scientists; WHO, World Health Organization
